# CD10 expression in the neuroendocrine carcinoma component of endometrial mixed carcinoma: association with long survival

**DOI:** 10.1186/s13000-016-0468-4

**Published:** 2016-02-01

**Authors:** Karina Uehara, Fukino Ikehara, Yasuka Tanabe, Iwao Nakazato, Mariko Oshiro, Morihiko Inamine, Takao Kinjo

**Affiliations:** Division of Morphological Pathology, Department of Basic Laboratory Sciences, University of the Ryukyus, 207 Uehara, Nishihara, Okinawa 903-0215 Japan; Department of Pathology, Okinawa Prefectural Nanbu Medical Center & Children’s Medical Center, 118-1 Arakawa Haebaru, Okinawa, 901-1193 Japan; Health Information Management Major, Faculty of International Studies, Meio University, 1220-1 Biimata, Nago, Okinawa 905-8585 Japan; Department of Medical Science of Woman and Reproduction, Graduate School of Medicine, University of the Ryukyus, 207 Uehara, Nishihara, Okinawa 903-0215 Japan

**Keywords:** Endometrial mixed carcinoma, Neuroendocrine carcinoma, Well differentiated endometrioid adenocarcinoma, CD10, PTEN, Long survival

## Abstract

**Backgound:**

Endometrial mixed carcinoma with the neuroendocrine carcinoma (NEC) component is rare and is believed to have a poor prognosis. CD10 expression is reported to be a favorable prognostic marker for some tumors such as B-lymphoblastic leukemia/lymphoma, but unfavorable for others. Here, we report the case of a 33-year-old woman diagnosed with endometrial mixed carcinoma with the NEC component expressing CD10 who showed a favorable outcome.

**Case presentation:**

The patient presented with lumbago and brownish discharge from the genitals. Imaging modalities revealed a large exophytic mass in the uterine corpus, and a small one in the uterine cervix. Radical hysterectomy with bilateral salpingo-oophorectomy was performed. Microscopic examination of the endometrial and cervical masses revealed that the NEC component accounted for the maximum area in both masses. However, small areas in both lesions showed well differentiated endometrioid adenocarcinoma (WDEA) components, and histological transition between the two components was also observed. In addition to CD56 and synaptophysin expression, the NEC component was positive for CD10 but negative for estrogen receptor (ER), progesterone receptor (PgR), and carcinoembryonic antigen (CEA). In contrast, the WDEA component expressed both ER and PgR, but neither CD10 nor neuroendocrine markers were demonstrated. The CD10 and neuroendocrine markers clearly distinguished between the NEC and WDEA components. Furthermore, retained expression of phosphatase and tensin homolog (PTEN) and weak phosphorylated Akt expression were found, which were assumed to suppress the aggressive behavior of the tumor. The patient received postoperative chemotherapy and has survived without recurrence for 6 years after the operation.

**Conclusion:**

This is the first case of endometrial mixed carcinoma with the NEC component expressing CD10 that showed a long survival.

## Background

Endometrial mixed carcinoma has recently been defined as a new entity of gynecological carcinoma. It is defined as a tumor composed of two or more histological types of endometrial carcinoma, at least one of which is of the type II category [1]. Most common mixed carcinomas consist of endometrioid and serous carcinoma components, however, the prevalence of mixed carcinoma with the neuroendocrine carcinoma (NEC) component is very rare [1, 2]. Among mixed carcinomas with the NEC component, the most common combination is with endometrioid adenocarcinoma (EA) [3, 4]. The prognosis of mixed carcinoma correlates with the highest-grade component [1]. Because the prognosis for endometrial small cell NEC and large cell NEC is poor [3, 4], it is considered that the prognosis of mixed carcinoma with the NEC component is unfavorable. Huntsman et al. reported the clinicopathological findings of 16 cases of small cell NEC, including 10 patients of mixed carcinoma with small cell NEC component. Among these 10 cases, 6 patients died between 2 months and 4 years postoperatively, 1 survived but exhibited multiple metastases [2]. Furthermore, Mulvany and Allen reported 5 cases of large cell NEC including 4 patients of mixed carcinoma with large cell NEC component showing that half of the mix carcinoma patients died within 2 years [5]. With regard to the association between clinical stage and prognosis of mixed carcinoma with small cell NEC component, Katahira et al. reviewed the literature and found that only 23 % of patients with International Federation of Gynecology and Obstetrics (FIGO) stage I died within 5 years, whereas 73 % of patients with stage II-IV died within 2 years [6]. Although the prognosis of endometrial mixed carcinoma with the NEC component is regarded as poor, especially advanced FIGO stage, very small number of long survival cases was reported [2, 7–9]. Half of the long surviving cases were early FIGO stage, however, the other factor (s) that may influence or correlate with favorable prognosis remain unclear.

CD10 is a cell-surface neutral endopeptidase and is distributed ubiquitously in various tissues. It is associated with multiple biological functions such as cellular proliferation, migration, differentiation and stem cell maintenance [10]. CD10 is expressed in several malignancies and through these functions CD10 is associated with biological properties of cancer including invasion, metastasis, and sensitivity to chemotherapy [10]. CD10 expression is a good prognostic marker in B-lymphoblastic leukemia/lymphoma [11], uterine cervical cancer [12], and non-small cell lung cancer [13]. On the other hand, it is a poor prognostic marker in several cancers such as gastric, pancreatic, colorectal carcinomas [14], melanoma [15], and skin cancers [16]. The significance of CD10 expression in endometrial mixed carcinoma with the NEC component is currently unknown.

Here we report a case of endometrial mixed carcinoma with the NEC component expressing CD10 at FIGO stage II. By immunohistochemistry, retained expression of phosphatase and tensin homolog (PTEN) and weak phosphorylated Akt expression were found in the NEC component, which may contribute to suppress the aggressive behavior of the tumor. The patient is alive without tumor recurrence for 6 years after surgery. To the best of our knowledge, this is the first case indicating the association between CD10 expression and favorable outcomes of mixed carcinoma with the NEC component.

## Case presentation

A 33-year-old woman presented with lumbago and brownish discharge from the genitals, which persisted for 3 months and continued to worsen. Physical examination and imaging modalities revealed exophytic tumors located in the uterine cervix and uterine corpus. As endometrioid carcinoma with neuroendocrine differentiation was suspected on the basis of preoperative biopsy, radical hysterectomy with bilateral salpingo-oophorectomy was performed. The patient received postoperative chemotherapy and has exhibited no recurrence for 6 years postoperatively.

## Methods

### Immunohistochemistry

Immunohistochemical staining was performed on formalin-fixed and paraffin embedded tissues. Four-micron sections were pretreated in citrate buffer (pH 6.0) in a microwave oven for antigen retrieval. The primary antibodies used in this study are shown in Table 1. The primary antibody reaction was performed at room temperature for 60 min, and the signal was detected using the LSAB 2 HRP system (DAKO) with liquid diaminobenzidine as the substrate chromogen.

**Table 1:** Primary antibodies used in this study

Marker	Source	Dilution	Clone/code
AE1/AE3	Dako	×800	M3515
34β-E12	Dako	×200	M0630
CAM 5.2	Ventana	diluted	CAM 5.2
EMA	Dako	×100	E29
CEA	Dako	×150	II-7
ER	Ventana	diluted	SP1
PgR	Ventana	diluted	1E2
CD10	Dako	×80	SS2/36
CD56	Novocastra	×100	B6
Chromogranin	Dako	×100	DAK-A3
Synaptophysin	Dako	×400	A0010
S-100	Dako	×2000	ER-PR8
p16	Abcam	×200	2D9A12
AKT1	Abcam	×100	ab54753
AKT1 (phospho S473)	Abcam	×100	104A282
PTEN	Abcam	×150	EPR9941
β-catenin	Millipore	×500	E247

## Results

### Gross findings

**Fig. 1:**
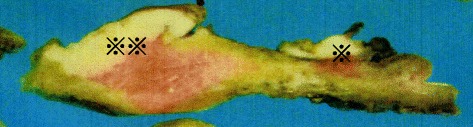
Macroscopic characteristics of the tumors. Bulky and solid tumors with whitish color are observed in the fundus (※※) and cervix (※). Large tumor (※※) arising from the endometrial mucosa of the fundus and invading the deep muscle layer. Small tumor (※) limited to the cervical mucosa, with no muscle layer invasion

The uterus along with both ovaries and fallopian tubes weighed 225 g. On sectioning, large bulky tumors were found in the uterine endometrium and uterine cervix (Fig. 1). The endometrial tumor measured 40 × 40 mm and showed deep invasion into the muscle layer. However, the serosal surface was not involved. The cervical tumor measured 28 × 28 mm and infiltrated into the uterine cervical stroma, but did not extend beyond the uterus. Based on these findings, the patient’s postsurgical stage was defined as FIGO stage II.

### Microscopic findings

**Fig. 2:**
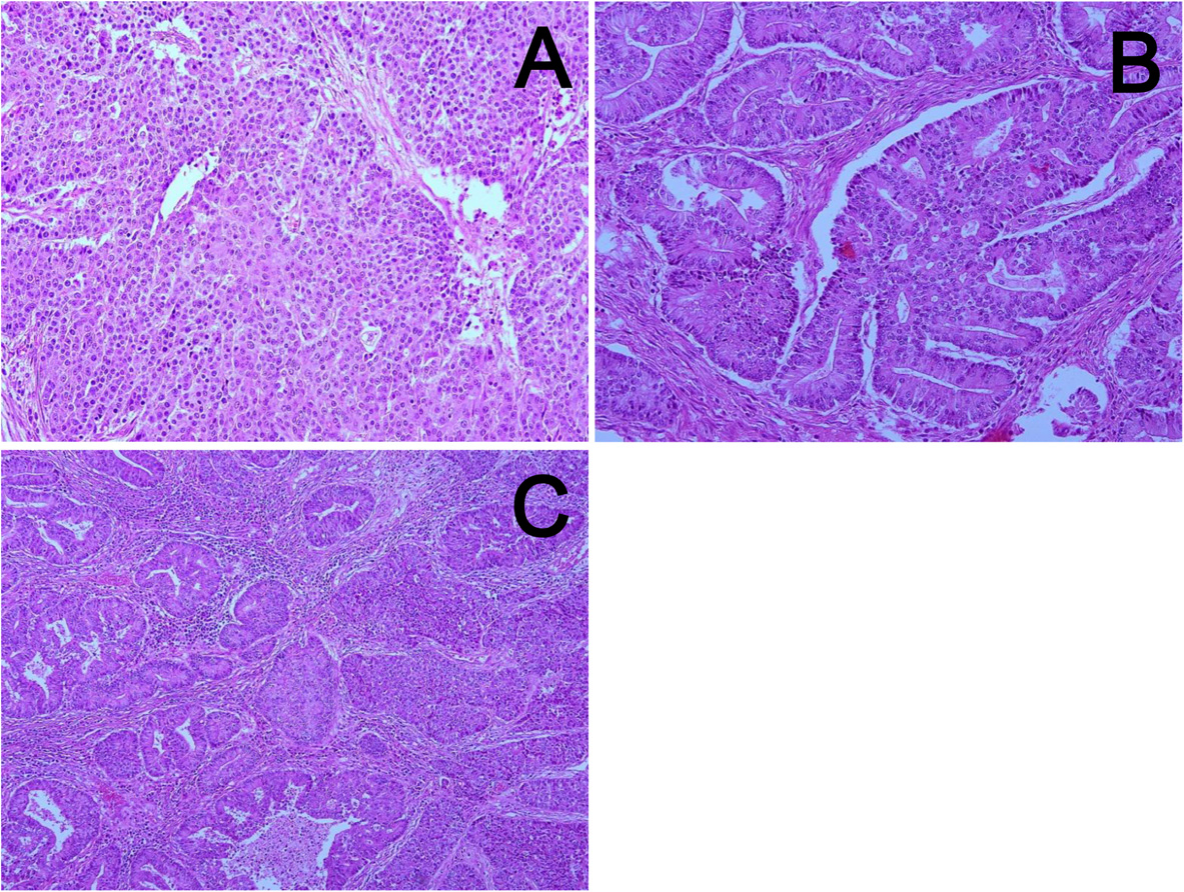
Microscopic findings of the tumors. **a**: The NEC component shows solid sheets and irregular gland like structures with vesicular nuclei and prominent nucleoli. Necrosis and rosette formation are noted. **b**: The WDEA component shows irregular tubular structures with cribriform pattern and complex folds. **c**: Histological transition is observed at the boundary between the NEC and WDEA components. Original magnification: **a**, **b**: ×200, **c**: ×100

Most of the bulky and ill-defined masses from the endometrium and cervix consisted of broad trabecular, solid sheets and irregular gland-like structures of cancer cells with vesicular nuclei, prominent nucleoli, and faintly eosinophilic cytoplasm. Peripheral palisading and central necrosis were apparent (Fig. 2a). These findings suggested NEC. Mitotic figures were conspicuous at 5–12 per 10 high-power fields (HPF). The NEC cells of the endometrial tumor invaded the myometrium to more than half its depth. The NEC cells of the cervical tumor infiltrated the cervical stroma, but did not reach the muscle layer. Lymphovascular invasion was observed, but no lymph node metastasis was identified. In small areas of the endometrial and cervical tumors, the cancer cells showed an atypical columnar shape with enlarged nuclei located on the basal side, irregular tubular glands showing a back-to-back structure, and complex fold formation. Solid nests of cancer cells occupied less than 5 % of both lesions (Fig. 2b). These findings corresponded with well differentiated endometrioid adenocarcinoma (WDEA). Histological transition was observed at the boundary between the NEC and WDEA components (Fig. 2c).

**Fig. 3:**
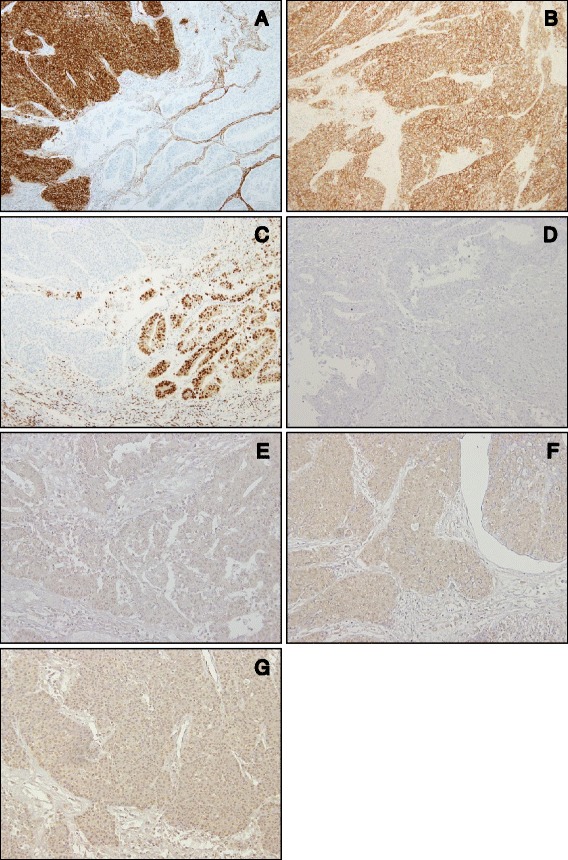
Immunohistochemical profile of the present case. **a**: The NEC component shows marked CD10 expression, whereas WDEA does not. **b**: CD56 immunoreactivity is observed in the NEC component. **c**: ER expression is not detected in the NEC component, but strong ER expression is demonstrated in the WDEA component. **d**: Neither the NEC component nor the WDEA component exhibits CEA expression. **e**: p16 is not noted in the WDEA component. **f**: PTEN expression is weak but diffusely detected in the NEC component. **g**: Phosphorylated AKT is detected faintly in the NEC component

Immunohistochemistry revealed that the NEC components in both endometrial and cervical tumors were strongly immunoreactive for CD10, positive for CD56, weakly positive for synaptophysin and p16, and negative for estrogen receptor (ER), progesterone receptor (PgR), carcinoembryonic antigen (CEA), and chromogranin (Fig. 3a-e). The NEC component was also positive for PTEN, comparable to the nuclear expression of stromal cells, whereas phosphorylated Akt was observed weakly in the cytoplasm (Fig. 3f, g). The WDEA components of both endometrial and cervical tumors were positive for ER and PgR, and negative for CD10, CD56, CEA, and p16 (Fig. 3a-e). The WDEA components of both tumors also exhibited PTEN expression and weak phosphorylated Akt expression. The results of the immunohistochemical examination are summarized in Table 2.

**Table 2:** Results of the immunohistochemical examination

	NEC	WDEA
AE1/AE3	+	+
34β-E12	-	+
CAM 5.2	++	++
EMA	+	+
CEA	-	-
ER	-	++
PgR	-	++
CD10	++	-
CD56	+	-
Chromogranin	-	-
Synaptophysin	+	-
S-100	-	-
p16	+	-
AKT1	+	+
AKT1 (phospho S473)	+	+
PTEN	+	+
β-catenin	-	-

NEC and WDEA indicate neuroendocrine carcinoma and well differentiated endometrioid adenocarcinoma, respectively

The intensity of immunohistochemical reactivity is expressed as ++representing strong intensity, + representing positive but not strong reactivity and, − representing negative

## Discussion

Because endometrial mixed carcinoma is a recently defined category of WHO tumor classification [1], previous studies reported the mixed carcinoma with NEC component as NEC associated other neoplasm in a series of small cell NECs or large cell NECs. We reviewed the literature and extracted 29 cases (Table 3). The mean age of patients was 61 years (from 30 to 88), and the most common histological combination was NEC and endometrioid adenocarcinoma. The present case is the second youngest and the longest survival case of this carcinoma. Among the extracted cases, 12 died within 3 years and only 5 cases, including our patient, had long-term survival of 3 years or more. These cases were treated with different methods of therapy. One case was treated only with surgery, two other cases underwent chemotherapy in addition to surgery, and the remaining cases were treated with a combination of surgery, radiation and chemotherapy. We could not find a common therapeutic regimen among the cases with long-term survival. Albores-Saavedra et al. reported 5 cases of polypoid small cell NEC including one mixed carcinoma with NEC component and suggested that polypoid endometrial NEC shows favorable prognosis [7]. In agreement with their observation, our case also showed bulky lesions of the endometrium and cervix, however, FIGO stage of the present case was more advanced than that of the previous case (FIGO II versus FIGO IA). Matsumoto et al. reported that the patient was still alive without the disease 4 years after the surgery despite recurrence, but the prognostic factors that contributed to the positive outcome were unknown [9].

**Table 3:** Clinical and pathological features of endometrial mixed carcinoma with NEC component in the lterature and in the present case

Case	Reference	Year	Age	Stage	Associated neoplasm	Treatment	Outcome	
1	Tohya et al. [30]	1986	64	IIIb	Endometrioid adenosquamous carcinoma	Surgery	AWD	3 months
2	Tenti et al. [31]	1989	70	IVb	Endometrioid adenocarcinoma	Surgery, Chemotherapy, Hormonal therapy	DOD	2 years
3	Campo et al. [32]	1992	72	UK	Poorly differentiated adenocarcinoma	Radiotherapy	DOD	6 months
4	Huntsman et al. [2]	1994	72	IIa	Endometrioid adenocarcinoma	Surgery, Radiotherapy	AWD	13 months
5	Huntsman et al. [2]	1994	55	IV	Endometrioid adenocarcinoma	Radiotherapy, Chemotherapy	DOD	12 months
6	Huntsman et al. [2]	1994	54	IVb	Atypical complex hyperplasia	Surgery	DOD	12 months
7	Huntsman et al. [2]	1994	30	IVb	Atypical complex hyperplasia	Surgery	LFU	
8	Huntsman et al. [2]	1994	37	IVb	Endometrioid adenocarcinoma	Surgery	LFU	
9	Huntsman et al. [2]	1994	70	IIa	Endometrioid adenocarcinoma	Surgery	DOD	1 year
10	Huntsman et al. [2]	1994	62	IIIa	Endometrioid adenocarcinoma	Surgery	DOD	2 months
11	Huntsman et al. [2]	1994	59	Ic	Endometrioid adenocarcinoma	Surgery, Radiotherapy, Chemotherapy	DOD	4 years
12	Huntsman et al. [2]	1994	58	IVb	Endometrioid adenocarcinoma	Surgery	DOD	4 months
13	Huntsman et al. [2]	1994	53	IIb	Endometrioid adenocarcinoma	Surgery	NED	2 months
14	van Hoeven et al. [22]	1995	59	I	Adenocarcinoma	Surgery	AWD	2 years
15	van Hoeven et al. [22]	1995	62	I	Adenocarcinoma	Surgery	AWD	1.5 years
16	Sekiguchi et al. [33]	1998	60	Ib	Adenocarcinoma, Squamous cell carcinoma	Surgery	DOD	28 months
17	Katahira et al. [6]	2004	54	Ib	Endometrioid adenocarcinoma, Squamous	Surgery, Chemotherapy	NED	28 months
					cell carcinoma			
18	Shaco-Levy et al. [34]	2004	79	UK	Papillary serous carcinoma	Surgery, Radiotherapy	DOD	5 months
19	Mulvany and Allen [5]	2007	80	Ic	Endometrioid adenocarcinoma	Surgery	DOD	5 months
20	Mulvany and Allen [5]	2007	77	IIb	Endometrioid adenocarcinoma	Surgery, Radiotherapy	DOD	23 months
21	Mulvany and Allen [5]	2007	79	IIIa	Endometrioid adenocarcinoma	Surgery, Radiotherapy	AWD	2 months
22	Mulvany and Allen [5]	2007	88	IIIc	Endometrioid adenocarcinoma	Surgery, Radiotherapy	AWD	1 month
23	Albores-Saavedra et al. [7]	2008	66	Ia	Endometrioid adenocarcinoma	Surgery	NED	4 years
24	Hwang et al. [35]	2010	59	Ic	Endometrioid adenocarcinoma, Squamous cell carcinoma	Surgery, Radiotherapy, Chemotherapy	NED	15 months
25	Sato et al. [8]	2010	56	IVb	Endometrioid adenocarcinoma	Surgery, Chemotherapy	DOD	3 years
26	Matsumoto et al. [9]	2011	44	IIIc	Atypical complex hyperplasia	Surgery, Radiotherapy, Chemotherapy	NED	4 years
27	Koo et al. [36]	2014	52	Ia	Endometrioid adenocarcnima, Atypical	Surgery, Chemotherapy	NED	15 months
					complex hyperplasia			
28	Koo et al. [36]	2014	63	Ib	Endometrioid adenocarcnima, Atypical	Surgery, Chemotherapy	NED	5 months
					complex hyperplasia			
29	Current report case	2015	33	II	Endometrioid adenocarcinoma	Surgery, Chemotherapy	NED	6 years

*UK* unknown, *DOD* dead on disease, *AWD* alive with disease, *NED* alive without evidence of disease, *LFU* lost to follow-up

The present case survived without tumor recurrence for 6 years after the surgery despite exhibiting FIGO stage II. As described earlier, the prognosis of endometrial mixed carcinoma is correlated with the highest grade component. The histological grading of NEC is an important prognostic factor in non-gynecological sites. Ki-67 labeling index and mitotic rate are currently used for the grading of neuroendocrine tumors (carcinoid, atypical carcinoid, small cell carcinoma, and large cell carcinoma), and these factors are well correlated with the prognosis [17, 18]. However, histological grading by use of the Ki-67 labeling index and mitotic rate for the uterine cervical NEC has not been demonstrated, because the prognostic outcome is poor regardless of the histological grading [19–21]. Although several studies have reported poor prognosis of endometrial NEC [2, 5, 6, 9, 22, 23], the effect of histological grade of NEC on the prognosis was not clarified. The present case showed 5–12 mitotic counts/ 10HPF and the ratio corresponds to neuroendocrine tumor grade 2 of the digestive system [24]. The relatively low counts of mitotic figures in our case may contribute to favorable prognosis, however, further studies are necessary to establish the significance of histological grade in NEC component in the endometrial mixed carcinoma.

The present case showed strong CD10 expression in the NEC component and also exhibited positive PTEN expression and weak phosphorylated Akt expression. With regards to molecular correlation between CD10 expression and favorable prognosis, CD10 is associated with cancer through its inhibitory effect on cell migration and proliferation [10]. The molecular mechanism of these effects induced by CD10 is associated with the cleavage and inactivation of fibroblast growth factor 2 (FGF2) [25] or direct binding with PTEN, leading to the enhancement of stability and activity, which result in the inhibition of the Akt pathway [26]. These inhibitory effects on cell migration and proliferation allow CD10 to prevent the aggressive behavior of tumor cells [10, 26]. Therefore, we assume that CD10 expression in the NEC component has an anti-cancer effect by retaining the expression of PTEN, which leads to decreased phosphorylated Akt expression. Although the NEC component is usually considered as a poor prognostic factor, the inhibitory effects of CD10 may have contributed to the favorable outcome seen in the present case.

We were unable to identify any other study that examined the correlation between CD10 expression in endometrial NEC and prognosis. As described earlier, CD10 is a favorable prognostic marker in some tumors such as B-lymphoblastic leukemia/lymphoma [27, 28] but unfavorable in others [14]. Intriguingly, the expression patterns of CD10 are correlated with the prognosis of renal clear cell carcinoma. The apical extracellular expression of CD10 shows better prognosis than cytoplasmic or intracellular membrane expression [29]. Because the prognostic value of CD10 may differ depending on the type of tissue from which the tumor arises, further studies are necessary to elucidate the significance of CD10 expression in prognosis.

## Conclusions

We reported a case of endometrial mixed carcinoma with NEC component showing favorable prognosis despite being FIGO stage II. Marked CD10 expression in the NEC component was associated with a retained expression of PTEN and a decreased expression of phosphorylated Akt, which may have resulted in anti-cancer effects and contributed to the favorable prognosis.

## Consent

Written informed consent was obtained from the patient for publication of this Case Report and any accompanying images. A copy of the written consent is available for review by the Editor-in-Chief of this journal.
